# Surrogate data analyses of the energy landscape analysis of resting-state brain activity

**DOI:** 10.3389/fncir.2025.1500227

**Published:** 2025-03-14

**Authors:** Yuki Hosaka, Takemi Hieda, Ruixiang Li, Kenji Hayashi, Koji Jimura, Teppei Matsui

**Affiliations:** ^1^Graduate School of Brain Science, Doshisha University, Kyotanabe, Japan; ^2^Department of Informatics, Gumma University, Maebashi, Japan

**Keywords:** resting-state fMRI, dynamic functional connectivity, energy landscape analysis, spontaneous activity, maximum entropy model

## Abstract

The spatiotemporal dynamics of resting-state brain activity can be characterized by switching between multiple brain states, and numerous techniques have been developed to extract such dynamic features from resting-state functional magnetic resonance imaging (fMRI) data. However, many of these techniques are based on momentary temporal correlation and co-activation patterns and merely reflect linear features of the data, suggesting that the dynamic features, such as state-switching, extracted by these techniques may be misinterpreted. To examine whether such misinterpretations occur when using techniques that are not based on momentary temporal correlation or co-activation patterns, we addressed Energy Landscape Analysis (ELA) based on pairwise-maximum entropy model (PMEM), a statistical physics-inspired method that was designed to extract multiple brain states and dynamics of resting-state fMRI data. We found that the shape of the energy landscape and the first-order transition probability derived from ELA were similar between real data and surrogate data suggesting that these features were largely accounted for by stationary and linear properties of the real data without requiring state-switching among locally stable states. To confirm that surrogate data were distinct from the real data, we replicated a previous finding that some topological properties of resting-state fMRI data differed between the real and surrogate data. Overall, we found that linear models largely reproduced the first order ELA-derived features (i.e., energy landscape and transition probability) with some notable differences.

## Introduction

Brain activity in the resting state, as measured using functional magnetic resonance imaging (fMRI) has been widely investigated for its potential applications in the non-invasive diagnosis of neuropsychiatric and neurological disorders (Fox and Raichle, 2007). A common assumption regarding the dynamics of resting-brain activity is that it can be explained by transitions between multiple brain states (Vidaurre et al., 2017; Noro et al., 2022; Hutchison et al., 2013; Calhoun et al., 2014; Preti et al., 2016). Recent studies have reported that dynamic features (e.g., brain states) extracted from measured resting-state brain activity can better explain subject-specific phenotypes (e.g., cognitive performance) than static features (Cabral et al., 2017; Liégeois et al., 2019), suggesting the potential importance of dynamic features for applications such as diagnosis of neuropsychiatric disorders.

However, it remains an open question whether the presence of multiple brain states is supported by resting-state fMRI data. Recent statistical examinations of common analysis techniques used to extract possible brain states from resting-state fMRI data, such as sliding-window correlation analysis (Hutchison et al., 2013; Matsui et al., 2018b) or co-activation pattern analysis (Liu et al., 2013), reported that these potential brain states can be fully reproduced with surrogate data which only have a single state by construction (Laumann et al., 2016; Liégeois et al., 2017; Matsui et al., 2022). Using surrogate data, these studies extensively examined the results obtained with sliding-window correlation analysis or co-activation pattern analysis (e.g., brain states, transition probability). These surrogate data were designed to retain selected statistical properties of the real fMRI data, such as covariance structure and autocorrelation, and were produced using stationary and linear models. Crucially, these methods produced almost identical results for real data and surrogate data, which contradicted the assumptions of sliding-window correlation analysis and co-activation pattern analysis (Laumann et al., 2016; Liégeois et al., 2017; Matsui et al., 2022). Thus, features extracted by sliding-window analysis or co-activation pattern analysis reflect stationary, linear properties of the real fMRI data, indicating that these analyses cannot be regarded as evidence of the non-stationarity, or multiple brain-states, of resting brain dynamics.

Given that methods based on sliding-window correlations and co-activation patterns are unable to extract dynamic features of resting-state fMRI data, a natural choice is to use alternative methods that do not use sliding-window correlations or co-activation patterns. Among these alternative methods, Energy landscape analysis (ELA) is a widely used approach inspired by statistical mechanical techniques developed for the analysis of Ising spins (Ezaki et al., 2017; Watanabe et al., 2014). On the basis of the maximum entropy principle, ELA recovers the energy landscape of resting-brain activity from the fMRI data, whose local minima correspond to the basins of attraction (i.e., brain states). Using the extracted energy landscape, ELA describes the dynamics of brain activity as transitions between brain states. Recent studies have reported that subject-level information (e.g., psychiatric conditions and cognitive scores) is reflected in the transition patterns among the states extracted by ELA (Watanabe and Rees, 2017; Kang et al., 2021). In the present study, we used surrogate data to examine statistical properties of the rs-fMRI data represented by the features extracted by ELA.

**Figure 1:**
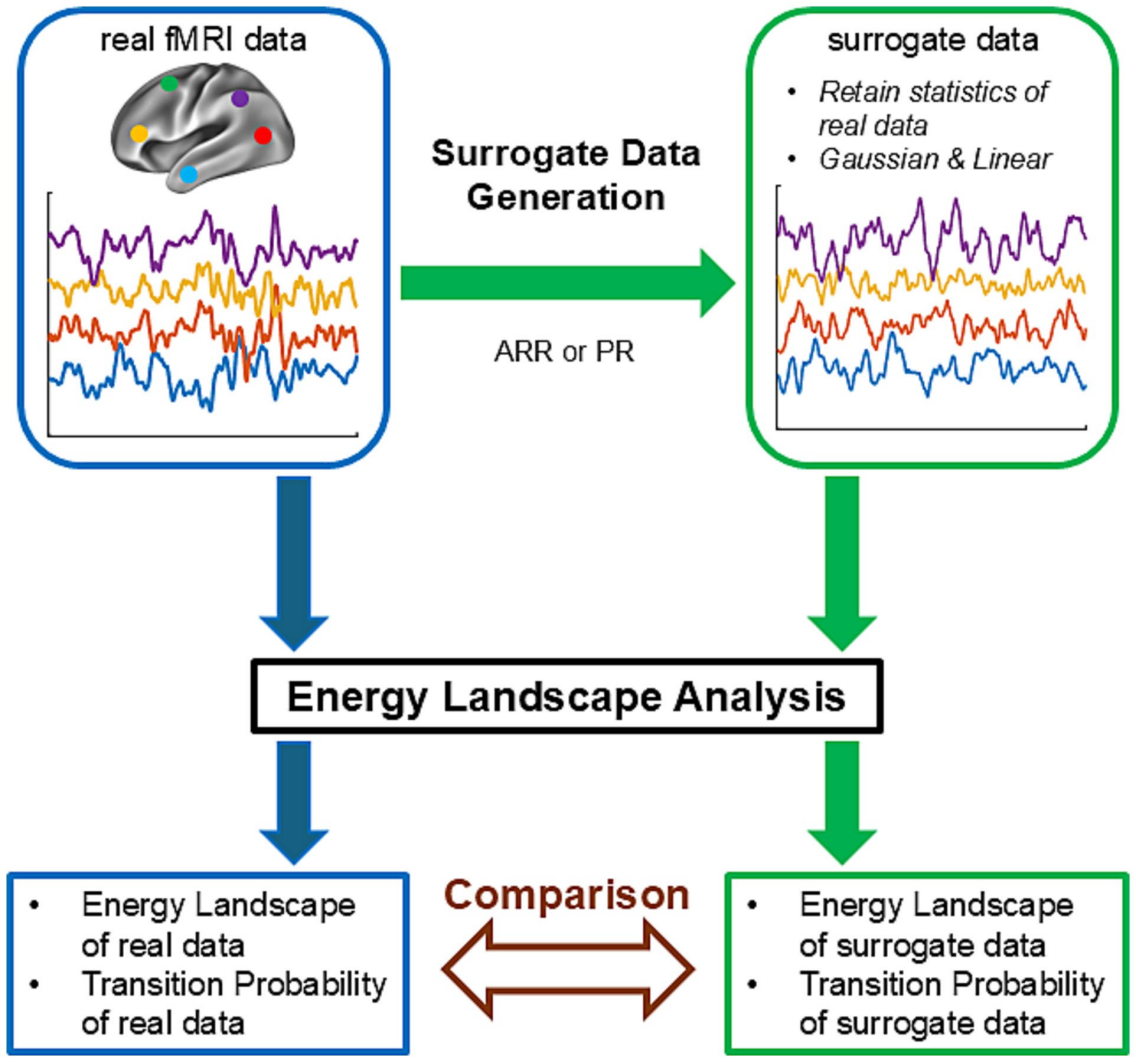
Schematics of surrogate data analysis. This schematic figure describes the strategy of the examination of ELA using surrogate data. The path indicated by blue arrows describes an ELA analysis of real resting-state fMRI data. The path indicated by green arrows describes an ELA analysis of surrogate data. In the first step of this path, surrogate time courses were constructed from the real fMRI time courses using autoregressive randomization (ARR) or phase randomization (PR). ELA was then applied to the surrogate data. Finally, in the step indicated by the brown bidirectional arrow, the results of the ELA (energy landscapes and transition probabilities) obtained in the two paths were compared.

Figure 1 illustrates the approach used in the present study. We first prepared real fMRI data of resting-state brain activity. ELA was applied to these data, yielding energy landscapes and transition probability matrices (a path indicated by blue arrows in Figure 1). Next, we generated surrogate data using real fMRI data and applied the same ELA to the surrogate data (a path indicated by green arrows in Figure 1). The surrogate data retained selected statistical properties, such as covariance structure, of the real fMRI data and were Gaussian and linear by construction. Finally, we compared the results of ELA obtained with the real data with those obtained with surrogate data (brown bidirectional arrow in Figure 1). Any difference between the two results could be attributed to statistical properties of the real data that were not used in the surrogate data, non-Gaussianity, non-linearity of the real data, or any combination of these factors.

## Materials and methods

### Dataset

We used the S1200 release of resting-state fMRI distributed by the Human Connectome Project (HCP (Van Essen et al., 2013).^1^ The data were preprocessed to obtain ROI-based timecourses [4,000 volumes × 264 ROIs × 1,002 subjects; repetition time (TR), 0.72 s; see Matsui et al., 2022, for details]. From 264 regions of interest (ROIs) defined in Power et al. (2011), we selected seven ROIs related to the cingulo-opercular network (CON), 11 ROIs related to the fronto-parietal network (FPN) and 12 ROIs related to the default mode network (DMN), whose centers were closest to the CON, FPN, and DMN ROIs defined in Fair et al. (2009). Because large amount of data is required to fit PMEM (Masuda et al., 2024), similar to Ezaki et al. (2017), we concatenated data from two participants (four scans per participants), yielding 501 real data samples in total.

### Generation of surrogate data

For each sample of real data, we applied three types of linear, stationary models to generate simulated data that retained certain statistical properties of the real data (Matsui et al., 2022; Liégeois et al., 2017). The first model retained only the covariance structure of the real data (Static Null). Simulated data for Static Null were generated using a multivariate Gaussian distribution with covariance matrices set to those of the real data. The second model was a first-order autoregressive randomization null model (ARR). The lag of the ARR null was set to 1. Thus, ARR assumed that the fMRI data at time *t* is the sum of the linear transformation (A*_1_*) of the fMRI data at time *t-1* and zero-mean multivariate Gaussian noise with a covariance matrix (*Σ*). The parameters for the autoregressive equation (*Σ, A_1_*) were fitted as described previously (Liégeois et al., 2017). Simulated data for ARR were generated using a randomly selected time point from the real fMRI data as the seed and by iteratively applying the autoregressive equation. The third model was a phase randomization null model (PR). PR retained the complete autoregressive structures of the real data as well as the covariance structures (Liégeois et al., 2017). Simulated data for the PR null were generated by first applying a discrete Fourier transform (DFT) to the real fMRI data. Random phases were then added to the Fourier-transformed data, and inverse DFT was applied. The added phases were independently generated for each frequency but were the same across brain regions (Liégeois et al., 2017). We referred to the simulated data produced by the null models as surrogate data.

### Energy landscape analysis

ELA was performed as described previously (Ezaki et al., 2017) using Matlab2023a (MathWorks, Natick, MA) with code provided by Ezaki et al. (2017). Briefly, for both real and surrogate data, fMRI time courses were binarized to −1 and 1. This implies that, for data with N ROIs, each volume could assume one of 2^N^ states. After binarization, a pairwise maximum entropy model was fitted to each sample of real or surrogate data, yielding an energy landscape. Basins of attraction of the energy landscapes were obtained by fitting dysconnectivity graphs (see Ezaki et al., 2017, for details).

### Comparison of ELA results obtained with real and surrogate data

Energy landscapes were compared by calculating Pearson’s correlation between an energy landscape of each sample of real data and an energy landscape obtained from the corresponding surrogate data.

For comparing the dynamics of real and surrogate data, we calculated a transition matrix describing the probability of switching (or staying) between basins of attraction. To obtain the transition matrix, each volume in the data was assigned to one of the basins of attraction, yielding a time course of state switching. Then, the probabilities of switching/staying from one basin to another in successive volumes were calculated. Comparisons of transition matrices were done in two methods. In the first method, we selected surrogate data with basins of attraction identical to those of the corresponding real data. An elementwise Pearson’s correlation was then calculated between the two matrices using all elements or only off-diagonal elements. In the second method, for each sample of surrogate data, we obtained the state-switching time course using basins of attraction obtained from the corresponding real data. We then calculated the transition matrix of the surrogate data and compared it with that of the real data using elementwise Pearson’s correlation (using all elements or only off-diagonal elements). Note that Pearson’s correlation using off-diagonal elements was calculated for those data that had more than three basins of attraction. For comparison of energy landscapes and transition probability, because each sample of surrogate data had a corresponding sample of real data, statistical testing was performed using a paired *t*-test.

### Topological data analysis

We conducted Mapper-based TDA following the procedures described by Saggar and colleagues with the Matlab codes provided by the researchers (Saggar et al., 2022). Briefly, in the first step, high-dimensional input data were embedded in a two-dimensional space using a filter function. To capture the intrinsic geometry of the data, we used a nonlinear filter function on the basis of neighborhood embedding. Specifically, Euclidian distances were calculated between all pairs of volumes. A k-nearest neighbor graph was then constructed using all volumes and calculated distances. Using the k-nearest neighbor graph, geodesic distances were calculated between all volumes in the input space. The geodesic distance was then embedded into a two-dimensional Euclidian space using multi-dimensional scaling. In the second step, overlapping two-dimensional binning was performed for data compression and noise reduction. Based on the previous study by Saggar et al. (2022), we chose a resolution parameter of 14. In the third step, partial clustering within each bin was performed. Finally, a shape-graph was generated by connecting nodes from different bins when any volumes were shared by the bins.

We randomly selected 100 HCP participants to match the sample size reported in the previous study (Saggar et al., 2022). For each participant, the time courses were concatenated across all four sessions. For statistical comparison, as in the previous study (Saggar et al., 2022), we calculated the proportion of high-degree nodes (degree>20). The statistical significance of the difference across real and surrogate data was assessed using one-way analysis of variance (ANOVA). Note that we applied the TDA using the same fMRI data and the same procedure for generating surrogate data as we used in the ELA. Thus, any difference between ELA and TDA could not be attributed to the difference in the data, the preprocessing procedures, or the generation of surrogate data.

### Data and code availability

The data used in this study are available from the website of (Ezaki et al., 2017) or from HCP. Code for reproducing essential results will be made available for download upon publication of the manuscript at https://github.com/teppei-matsui/EL. All codes used for the analysis will be provided upon reasonable request to the corresponding author.

## Results

### Energy landscape reflects the covariance structure of resting-brain activity

**Figure 2:**
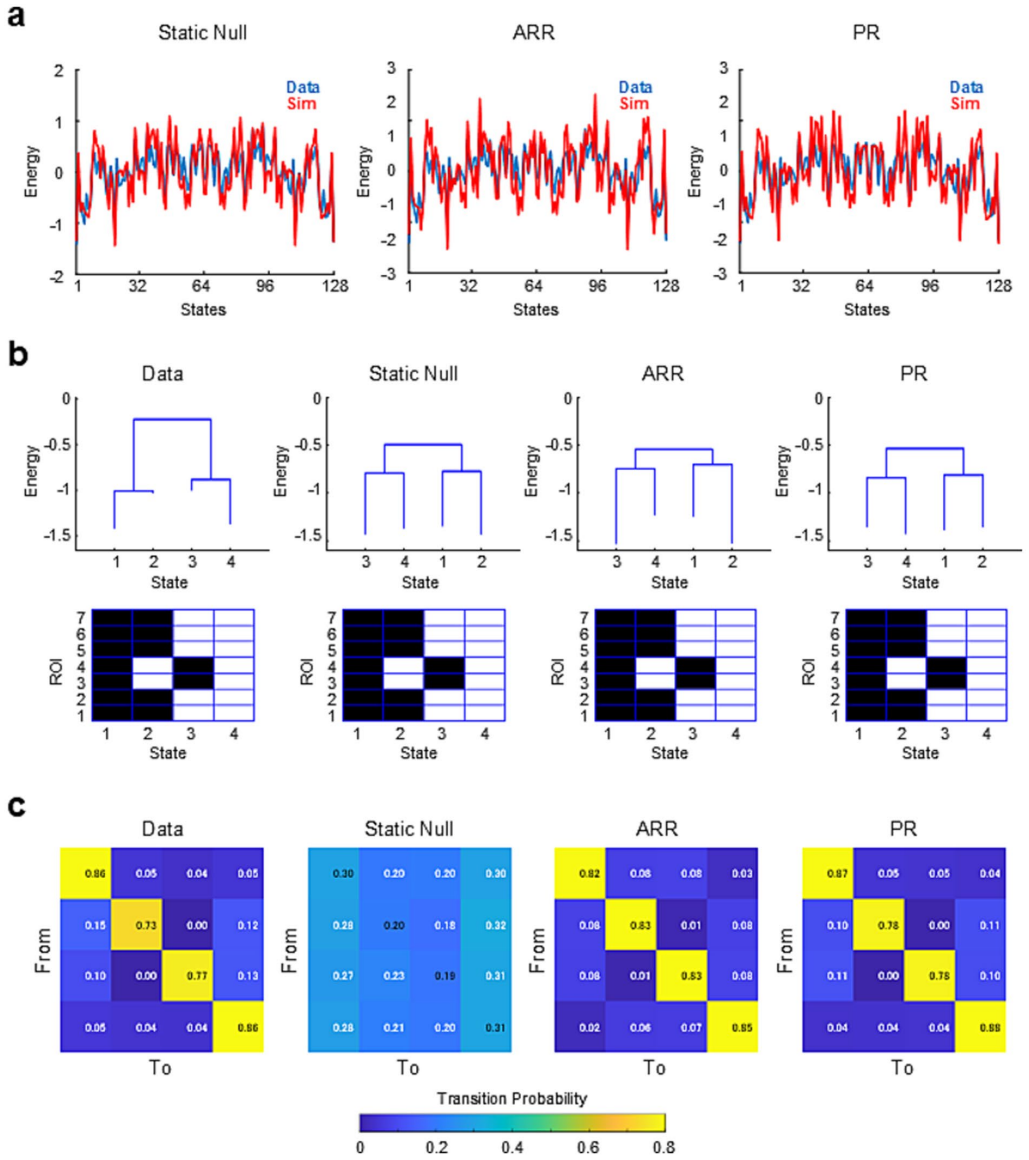
Energy landscapes and transition probability matrices for example and surrogate data calculated from it. **(A)** Plots of energy landscapes. Energy landscapes for real data (blue) and surrogate data (red) are overlaid for each type of surrogate data. **(B)** Dysconnectivity graphs (top) and activity patterns of states corresponding to the local minima (bottom) for real and surrogate data. **(C)** Matrices of transition probability between energy minima (basins). Note that energy minima were identical for the real data and all surrogate data.

First, we compared the energy landscapes obtained with real data and surrogate data constructed by stationary null models. Figure 2A shows the energy landscapes of example real data and the surrogate data constructed from it. This example demonstrates that the energy landscapes of the real and surrogate data were highly correlated and largely overlapped for all the null models tested (Static Null, R = 0.845; ARR, R = 0.827; PR, R = 0.835) (Figure 2A). These results indicate that linear and stationary models taking into account the second-order statistics of the data are enough to capture the shape of the energy landscape.

In contrast to energy landscapes, there were some notable differences in disconnectivity graphs for the real and surrogate data (Figure 2B). The energy barrier between states 1/2 and 3/4 is greatly reduced in the surrogate data. Similarly, in the real data, states 2 and 3 appear as minor basins, whereas in the surrogate data these basins become large. The discrepancy in disconnectivity graphs was unexpected given PMEM is designed to fit correlation structures in the data that were preserved in surrogate data.

To examine the source of the difference in dysconnectivity graphs, we compared the covariance matrices of the real rs-fMRI data and those of the surrogate data. To reduce the influence of sampling error due to random simulation, we generated 100 times more time points for surrogate data compared to the real data. We found that the covariance matrices of real and surrogate data produced by static null, ARR and PR nearly perfectly matched (Supplementary Figure 1A), confirming the expectation that these null models preserve the covariance of the real data. We next examined covariance matrices obtained using the real and surrogate data after the binarization. Because PMEM only takes binary inputs, both real and surrogate data were binarized before fitting PMEM (Masuda et al., 2024). Importantly, we found that the covariance matrices of the real and surrogate data became dissimilar after the binarization (Supplementary Figure 1B). Together, these results suggest that the binarization of the data caused a mismatch in real and surrogate matrices which in turn caused the discrepancy in disconnectivity graphs.

To further examine the possibility that binarization caused the discrepancy of the real and surrogate disconnectivity graphs, we next created new static null surrogate data of the binarized real rs-fMRI data using a method for generating multivariate binary sequences with specified covariance structure (Macke et al., 2009). Notably, the new binary surrogate data revealed near perfect matchings of the energy landscape and the disconnectivity graph with those of the real data (Supplementary Figures 2A,B). Moreover, the basins of attraction also perfectly matched (Supplementary Figure 2C). These results indicate that linear and stationary model taking into account the mean and the covariance of the binarized real data was sufficient to accurately capture the shape of the energy landscape and the dysconnectivity graph. Moreover, the results suggest that binarization of data is likely to be a major cause of the discrepancy between the real and surrogate disconnectivity graphs seen in Figure 2B.

Because the purpose of present study is to understand the statistical features of (non-binarized) rs-fMRI data extracted by ELA features, the rest of the paper used surrogate data generated using non-binarized real data.

### Transition patterns among states reflect the autocorrelation of resting-brain activity

Next, we examined whether the transition patterns among the energy minima could be captured by the null models. We found that the local minima in the energy landscapes were identical for the real and surrogate data. Note that the basins of attraction differed as suggested by the difference between real and surrgoate disconnectivity graphs (Figure 2B). Figure 2C shows the transition probability matrices describing the state-switching dynamics of the real and surrogate resting brain activities. Unlike the shape of the energy landscape, transition probabilities obtained with Static Null showed low correlations with those obtained with the real data (R = 0.138). In contrast, the transition probabilities obtained with ARR and PR showed extremely high correlations with the real transition probabilities (ARR, R = 0.992; PR, R = 0.999). The correlation between transition matrices was higher for PR than for Static Null even when excluding diagonal elements (Static Null, R = 0.773; PR = 0.953). Though this correlation value was slightly smaller for ARR than Static Null (ARR, R = 0.697), actual correlation values were much closer to those of the real data in ARR than Static Null (Figure 2C). The relatively high positive correlation for Static Null was likely due to the relative frequency of states reflected in the covariance structure. High self-transition probabilities in the real data and ARR/PR surrogate may reflect the temporal sampling rate (TR) of fMRI scanning in the HCP dataset (0.72 s) that is substantially faster than typical temporal autocorrelation of fMRI signal. These results indicate that the dynamics of the transitions between energy minima can be effectively captured by linear autoregressive models.

### Test of reproducibility in a large database

**Figure 3:**
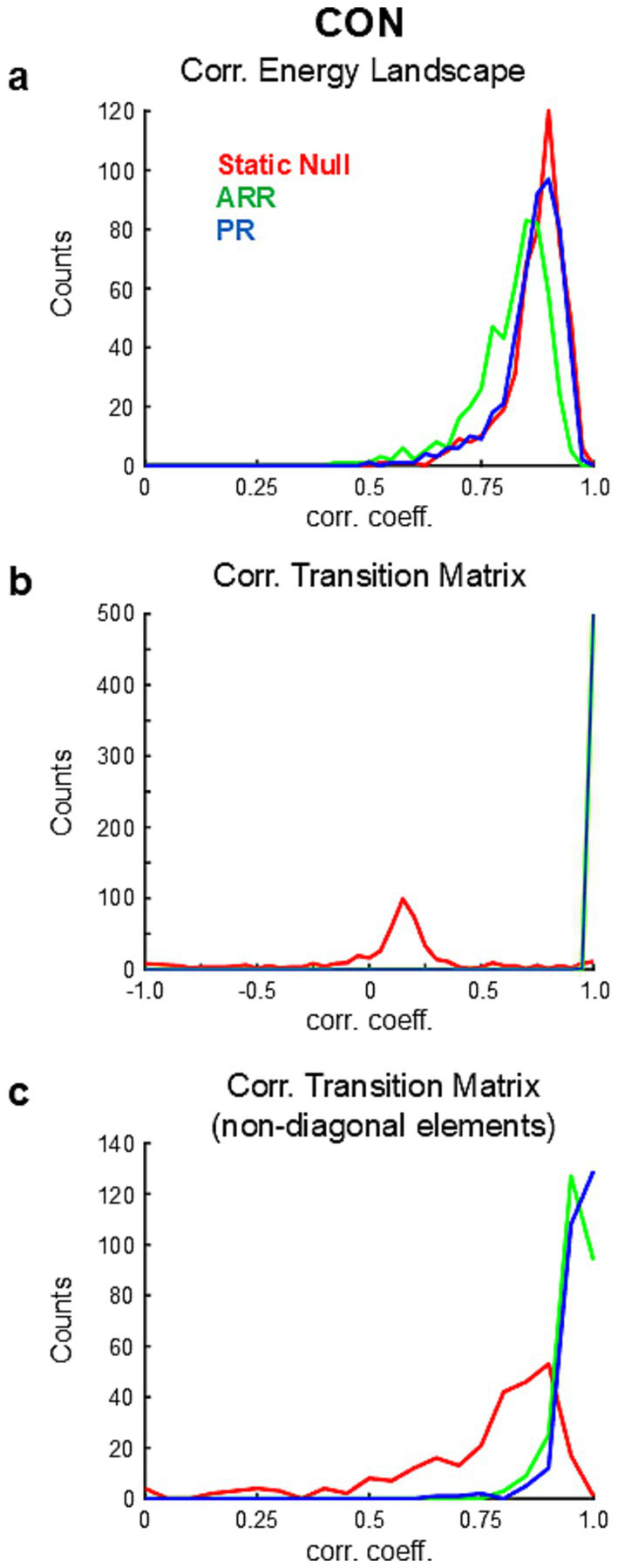
Results of population analysis based on HCP. **(A)** Distribution of correlation coefficients between energy landscapes calculated from real and surrogate data. **(B)** Distribution of correlation coefficients between transition matrices calculated from real and surrogate data. Note that the correlations were calculated using all elements of the transition matrices. **(C)** Distribution of correlation coefficients between transition matrices calculated from real and surrogate data using only non-diagonal elements of the transition matrices.

To confirm whether these observations also hold in other datasets, we compared energy landscapes of real and surrogate data using a publicly available large-scale database of resting-state fMRI provided by HCP. From this database we obtained 501 samples of CON activities. Correlation coefficients of energy landscapes of the real and surrogate data were high for all tested null models (Figure 3A). Although the differences were small, the correlation values were the highest for Static Null and lowest for ARR [Static Null, 0.871 ± 0.068 (mean ± s.d.); ARR, 0.818 ± 0.085; PR, 0.864 ± 0.070; *p* < 0.10^−10^ (uncorrected) for all pairwise comparisons, paired *t*-test]. These results suggest that the shape of the energy landscape of resting-state fMRI data can be largely captured by stationary and linear statistical properties.

A potential concern is whether the estimations of energy landscapes were reliable enough to distinguish between energy landscape from different samples (Note that different samples consist of data from different participants; see Methods for details). To address this, we calculated the correlations of ELs between different samples from the HCP dataset for all three networks (i.e., CON, FPN, DMN). We then compared these correlation values with those obtained by comparing energy landscapes from real versus surrogate data. The correlation values of energy landscapes between samples were R = 0.478 ± 0.194 (mean ± SD) for CON, R = 0.463 ± 0.114 for FPN and R = 0.358 ± 0.138 for DMN. These correlation values were significantly lower than those between real and surrogate data within the same sample, suggesting that the estimation of energy landscape was reliable enough to distinguish between energy landscapes obtained from different samples (participants). Note that, because data from different participants were combined to meet the data requirement of PMEM (Ezaki et al., 2017), these results may be better interpreted that the energy landscape representing inter-subject variability in fMRI signals can be reproduced by linear models.

Additionally, we examined whether energy landscapes from the same network (two ELs from CON) are more similar to each other than energy landscapes from different networks (CON and FPN). Because two energy landscapes can be compared only when the two sets of ROIs have one-to-one correspondence, we selected the first seven ROIs in FPN to match the numbers of ROIs in the two networks. Note that the correspondence between the ROIs of CON and the selected ROIs of FPN was arbitrary. Given this set of ROIs, we found that the correlations between energy landscapes from CON (R = 0.476 ± 0.192, mean ± SD) were significantly higher than those between energy landscapes from CON and FPN (R = 0.350 ± 0.183) (*p* ≪ 0.001, two-sample *t*-test, N = 125,250 pairs of samples) (Supplementary Figure 3). These results suggest that the energy landscape captures common network-specific characteristics across subjects. Furthermore, given the similarity of the energy landscapes from different samples (but the same network) was substantially smaller than that between real and surrogate energy landscapes (R > 0.75), it is conceivable that the energy landscape also captures sample-specific characteristics.

For simulations yielding identical energy landscapes (32, 17, and 34 out of 501 simulations for Static Null, ARR, and PR, respectively), correlation coefficients between transition matrices obtained from data and simulations were high for ARR [R = 0.994 ± 0.006 (mean ± s.d.) with diagonal elements; R = 0.751 ± 0.311 without diagonal elements] and PR (R = 0.992 ± 0.011 with diagonal elements; R = 0.711 ± 0.417 without diagonal elements) but not for Static Null (R = 0.113 ± 0.125 with diagonal elements; R = 0.545 ± 0.532 without diagonal elements).

**Figure 4:**
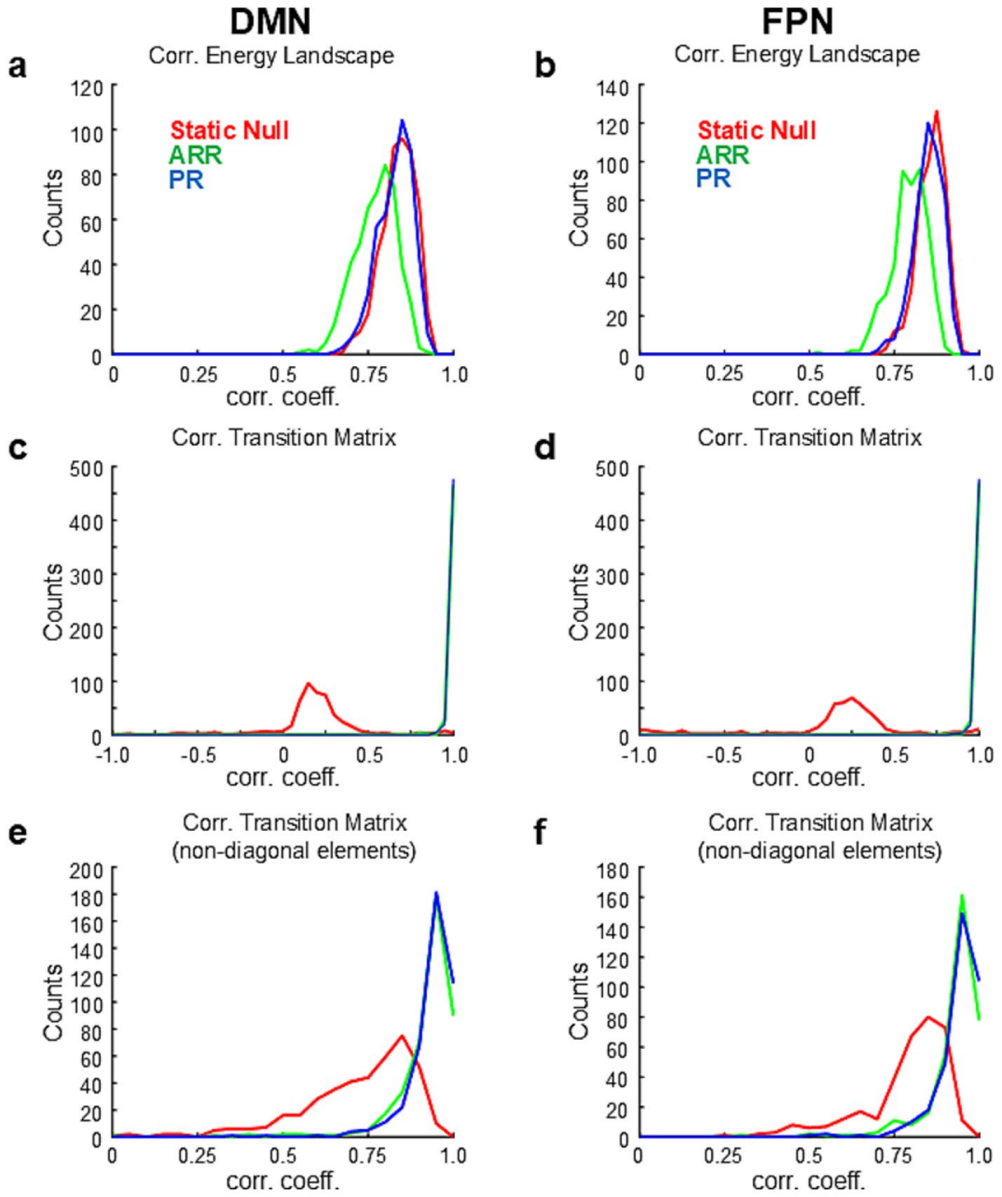
Results of population analysis based on HCP for DMN and FPN. **(A)** Same as Figure 3 but for DMN and FPN. **(A,B)** Distribution of correlation coefficients between energy landscapes for DMN **(A)** and DMN **(B)**. **(C,D)** Distribution of correlation coefficients between transition matrices for DMN **(C)** and FPN **(D)**. **(E,F)** Distribution of correlation coefficients between transition matrices for DMN **(E)** and FPN **(F)**.

To further examine the similarity of dynamics between data and simulations, based on the high similarities of energy landscapes between them (Figure 3A), we calculated transition probability matrices in each sample of surrogate data using the basins of attraction calculated from the corresponding real data. Transition matrices calculated with surrogate data were highly correlated with those calculated with ARR (R = 0.999 ± 0.002 with diagonal elements) and PR (R = 0.999 ± 0.003 with diagonal elements) but not with Static Null (R = 0.096 ± 0.390 with diagonal elements) (Figure 3B; Supplementary Figure 4). No significant difference was found between ARR and PR [*p* > 0.015 (uncorrected), paired *t*-test]. Correlations calculated only using non-diagonal elements in ARR and PR were still higher than those in Static Null (Figure 3C), although Static Null had positive overall correlations (R = 0.747 ± 0.203 without diagonal elements). Though the difference was small, correlation values were significantly higher for PR (R = 0.956 ± 0.037 without diagonal elements) than ARR (R = 0.963 ± 0.043 without diagonal elements) [*p* < 0.004 (uncorrected), paired *t*-test]. Similar results were obtained for DMN and FPN (Figure 4). Overall, these results confirmed the reproducibility of the observation made with the example data (Figure 2).

### Topological analysis confirmed the distinction between real and surrogate data

All of the results presented so far indicated that ELA yields largely identical outcomes for real resting-state fMRI data and surrogate data. This raises the possibility that real resting-state fMRI data are indeed fully describable by linear autoregressive models with residuals that have Gaussian distributions. However, previous studies reported that TDA can distinguish between real fMRI data and Gaussian, linear surrogates (Saggar et al., 2022; Geniesse et al., 2022). Thus, we conducted Mapper-based TDA (Saggar et al., 2022) to ensure that the real resting-state fMRI data contained features that were not captured by the surrogate data.

**Figure 5:**
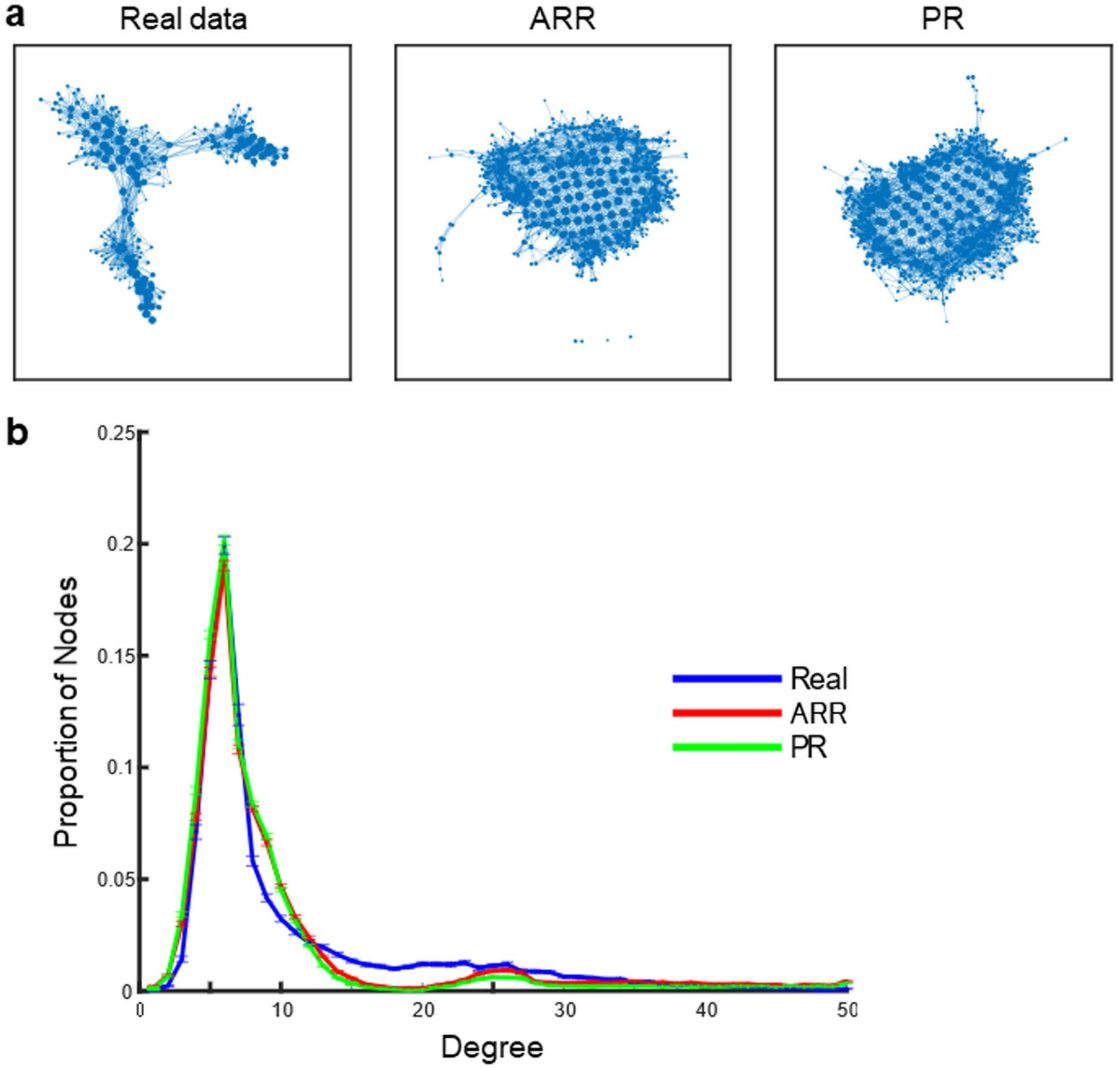
Topological landscape of real and surrogate data. **(A)** Mapper shape graph of real resting-state fMRI data of an example participant. A Mapper shape graph of ARR surrogate data constructed using the real data in **(A)**. A Mapper shape graph of PR surrogate data constructed using the real data in **(A)**. **(B)** Distributions of the degree of Mapper shape graphs across HCP participants. Error bars, SEM.

Figure 5A shows example topological landscapes of the real resting-state fMRI data and ARR and PR surrogates as visualized by Mapper-generated shape graphs (Figure 5A). Mapper-generated shape graphs of the real data showed segregation of nodes to multiple clusters. In contrast, nodes in the shape graphs of ARR and PR surrogates showed a single homogeneous cluster and appeared distinct from the real shape graphs. To quantify the difference of the graph-structure, we calculated nodal degree distributions (Figure 5B). Compared with ARR and PR, the real data contained nodes with high degree at a higher proportion, consistent with the previous study (Saggar et al., 2022). To test statistical significance, we assessed statistical differences in the proportion of high-degree nodes in the real versus surrogate data. Using the same threshold for high-degree node (>20) as in the previous study (Saggar et al., 2022), we found statistically significant differences across real and surrogate data [*F*(2, 299) = 75.49, *p* < 3.10 × 10^−27^]. These results confirmed previous reports that topological landscapes represent features of the real resting-state fMRI data that are not captured by Gaussian, linear surrogates (Saggar et al., 2022; Geniesse et al., 2022). Moreover, the results suggest that the energy landscapes obtained with ELA do not capture these features.

## Discussion

The current results revealed that two key results of ELA, the energy landscape and transition matrices describing the state-switching dynamics, can be explained by the stationary null models taking into account the covariance and the autocorrelation, respectively, of real resting-brain activity data. The finding that Static Null reproduced the energy landscape suggests that the energy landscape reflects the covariance structure of the resting-state fMRI data. The absence of a significant difference between ARR and PR for explaining the transition matrix suggests that first-order autocorrelation is sufficient to explain the state-switching dynamics.

The purpose of the present study is to examine and specify which statistical features of the resting-state fMRI data are being extracted by PMEM-based ELA. To achieve this, we analyzed how ELA interprets both real data and surrogate data generated by linear models. Since PMEM is a function that fits the correlations between ROIs up to the second order, and the energy landscape requires only that information, it is not surprising to observe that PMEM-based ELA produces very similar energy landscapes for the real data and surrogate data which preserves the correlation structure. Importantly, even if the data are shuffled, the same energy landscape can be obtained. Therefore, by construction, the energy landscape obtained using PMEM is agnostic to the temporal structure of the data. In other words, the energy landscape obtained by PMEM-based ELA does not necessarily specify the dynamics of the data. Hence, it remains unclear which feature of the dynamics of resting-state fMRI data is captured by the state-transition probability of ELA. This is one of the main questions of the study that we addressed based on the series of surrogate data analyses. Nevertheless, since several previous studies have shown that the first-order autoregressive model can well describe the dynamics of resting-state fMRI data (Liégeois et al., 2017; Matsui et al., 2022; Nozari et al., 2024), it is expected that data preserving first-order temporal structure and correlations preserve the transition probability obtained by ELA. The present study confirmed this expectation quantitatively. It should also be noted that some of the similarity in transition patterns of the real and surrogate data may be attributed to the property that transitions can only occur between activity patterns in adjacent basins.

Fundamentally, PMEM-based ELA is a technique that labels states on a map of brain activity based on a single probability distribution, and then examines whether the indices derived from such labeling can capture features of brain dynamics. Given this perspective, the present study aimed to specify the statistical features mapped to the ELA feature by examining whether a particular type of surrogate data replicated an ELA feature of the real data. An important point about this mapping is that the energy landscape does not utilize temporal information of the data: an energy landscape remains invariant under temporal shuffling of the data. Thus, in PMEM-based ELA, whereas spatial components (i.e., local minima) are derived from the energy landscape, the transition matrix and trajectories of states are not directly derived from the energy landscape itself. Rather, transition matrices and trajectories are projections, or mappings, of the real dynamics onto a small number of spatial components. Thus, although ELA interprets the resting-brain activity as movements between local minima, ELA does not strongly claim that each basin corresponds to an “actual” brain state, particularly since there is no ground truth of the actual brain states for resting-state data. In this sense, ELA can be viewed similarly to clustering. The possibility that the energy landscape obtained by PMEM-based ELA does not contain information about temporal structure of the data is supported by a previous study that analyzed data from cultured neurons (Yeh et al., 2010). Interestingly, this study also showed that an extension of PEME by incorporating additional terms representing temporal correlation successfully recapitulated spatiotemporal sequences of multi-neuronal spikes (Yeh et al., 2010). Nevertheless, the present results do not preclude a possibility that brain dynamics are driven by state switching, and a possibility that the current form of PMEM-based ELA can map some portion of such state-switching features as trajectories in the energy landscape.

One of the limitations of the PEME-based ELA is that PEME-based ELA can only handle small number of ROIs at a time. This is due to the large amount of data required to fit PMEM (see a recent tutorial; Masuda et al., 2024, for details). Because PMEM-based ELA can only analyze a small number of ROIs, previous studies using this method have typically used ROIs within well-known particular networks (e.g., DMN, FPN) (Watanabe et al., 2014; Ezaki et al., 2017). Thus, unlike whole-brain analyses that extract global brain-states such as DMN-or FPN-dominant states, the brain-states extracted by ELA may be considered as substates within a global brain-state, making it difficult to directly compare these two types of brain-states.

Another important characteristic of the PMEM-based ELA is that it requires binarization of the data. We found that the differences of real and surrogate energy landscapes and disconnectivity graphs are likely due to binarization of the data. A limitation of the present study is that we do not have a clear mathematical understanding of the statistical features being emphasized by the binarization and the subsequent procedures of ELA. Nevertheless, this newly found characteristics of PMEM-based ELA is a potentially useful characteristic which may be exploited in the future research.

Comparison of ELA and TDA revealed the existence of features of the real resting-state fMRI data not captured by Gaussian, linear surrogates. Consistent with previous studies (Geniesse et al., 2022; Saggar et al., 2022), we found that topological landscapes could distinguish between real resting-state fMRI data and surrogate data produced by linear, Gaussian models. The topological features do not necessarily reflect dynamic aspects of the data, because TDA-mapper did not use temporal information (i.e., the same topological landscapes would be obtained for temporally shuffled data). Further characterization of the topological features obtained by TDA-mapper will be described elsewhere.

It should be noted that the fact that surrogate data produced by a linear autoregressive model preserved the energy landscapes and transition probabilities of the real data does not diminish ELA’s utility in describing resting-brain activity. Additionally, our statement that linear models replicate the energy landscape and dynamics of the resting-state brain activity is a mathematical but not a conceptual one. Without the concept of the energy landscape, the definition (or concept) of basins is obscured. The concepts of basins and ELA could be useful to obtain intuitive pictures of resting-state fMRI data which are otherwise high-dimensional and complex. For example, Ezaki et al. (2018) discuss the number and efficiency of switching between two distant states, and Watanabe and Rees (2017) show that indirect transitions through minor states are useful in describing dynamics. ELA is useful for capturing these overall trajectories in an intuitive picture. An important issue is the extent to which linear models preserve the trajectory properties. One limitation of the present study is that only the first-order trajectory (i.e., transition probability) was tested. Unless all the temporal features of the real resting-state fMRI data are perfectly reproducible by linear models, there remains a possibility of finding trajectories that deviate or cannot be understood as mappings of statistical properties described by linear models. The present results, nevertheless, indicate that the results obtained by ELA, in particular the brain-states and transition probability, should be interpreted with care (see Matsui and Yamashita, 2023, for related discussions).

From a broader perspective, the present results align with a recent proposal that macroscopic resting brain activity is best described with linear models (Nozari et al., 2024). Taken together with previous studies (Laumann et al., 2016; Liégeois et al., 2017; Matsui et al., 2022), the present findings indicate that the dynamics of resting-state fMRI which resemble state-switching dynamics can be well described by simple linear models. An alternative possibility is that, because of the large amount of measurement noise in fMRI, many existing analysis methods, such as ELA, cannot extract nonlinear and complex dynamics (e.g., state-switching) in fMRI data. To distinguish between these possibilities and determine the extent to which simple models describe macroscopic resting-brain dynamics, future animal studies using measurements with higher signal-to-noise ratio, such as calcium imaging, would be useful (Li et al., 2023; Matsui et al., 2018a).

## Conclusion

Using surrogate data analyses, we found that the features of resting-state fMRI activity extracted by ELA, namely the shape of the energy landscape and the transition patterns among the energy minima, can be largely explained by stationary and linear statistical properties of the data. This finding supports the notion that resting-state fMRI activity is well described by linear models.

## Data Availability

Publicly available datasets were analyzed in this study. This data can be found at: Human Connectome Project.

## Footnotes

^1^ http://humanconnectomeproject.org/

## References

[ref1] Cabral J. Vidaurre D. Marques P. Magalhães R. Silva Moreira P. Miguel Soares J.. (2017). Cognitive performance in healthy older adults relates to spontaneous switching between states of functional connectivity during rest. Sci. Rep. 7:5135. doi: 10.1038/s41598-017-05425-7, PMID: 28698644 PMC5506029

[ref2] Calhoun V. D. Miller R. Pearlson G. Adalı T. (2014). The Chronnectome: time-varying connectivity networks as the next frontier in FMRI data discovery. Neuron 84, 262–274. doi: 10.1016/j.neuron.2014.10.015, PMID: 25374354 PMC4372723

[ref3] Ezaki T. Sakaki M. Watanabe T. Masuda N. (2018). Age-related changes in the ease of dynamical transitions in human brain activity. Hum. Brain Mapp. 39, 2673–2688. doi: 10.1002/hbm.24033, PMID: 29524289 PMC6619404

[ref4] Ezaki T. Watanabe T. Ohzeki M. Masuda N. (2017). Energy landscape analysis of neuroimaging data. Philos. Trans. A Math. Phys. Eng. Sci. 375:20160287. doi: 10.1098/rsta.2016.0287, PMID: 28507232 PMC5434078

[ref5] Fair D. A. Cohen A. L. Power J. D. Dosenbach N. U. Church J. A. Miezin F. M.. (2009). Functional brain networks develop from a “local to distributed” organization. PLoS Comput. Biol. 5:e1000381. doi: 10.1371/journal.pcbi.1000381, PMID: 19412534 PMC2671306

[ref6] Fox M. D. Raichle M. E. (2007). Spontaneous fluctuations in brain activity observed with functional magnetic resonance imaging. Nat. Rev. Neurosci. 8, 700–711. doi: 10.1038/nrn2201, PMID: 17704812

[ref7] Geniesse C. Chowdhury S. Saggar M. (2022). Neumapper: a scalable computational framework for multiscale exploration of the Brain’s dynamical organization. Netw. Neurosci. 6, 467–498. doi: 10.1162/netn_a_00229, PMID: 35733428 PMC9207992

[ref8] Hutchison R. M. Womelsdorf T. Allen E. A. Bandettini P. A. Calhoun V. D. Corbetta M.. (2013). Dynamic functional connectivity: promise, issues, and interpretations. Neuroimage 80, 360–378. doi: 10.1016/j.neuroimage.2013.05.079, PMID: 23707587 PMC3807588

[ref9] Kang J. Jeong S. O. Pae C. Park H. J. (2021). Bayesian estimation of maximum entropy model for individualized energy landscape analysis of brain state dynamics. Hum. Brain Mapp. 42, 3411–3428. doi: 10.1002/hbm.25442, PMID: 33934421 PMC8249903

[ref10] Laumann T. O. Snyder A. Z. Mitra A. Gordon E. M. Gratton C. Adeyemo B.. (2016). On the stability of bold FMRI correlations. Cereb. Cortex 27, 4719–4732. doi: 10.1093/cercor/bhw265, PMID: 27591147 PMC6248456

[ref11] Li R. Ohki K. Matsui T. (2023). Ketamine-induced 1-Hz oscillation of spontaneous neural activity is not directly visible in the hemodynamics. Biochem. Biophys. Res. Commun. 678, 102–108. doi: 10.1016/j.bbrc.2023.08.034, PMID: 37625269

[ref12] Liégeois R. Laumann T. O. Snyder A. Z. Zhou J. Yeo B. T. T. (2017). Interpreting temporal fluctuations in resting-state functional connectivity MRI. Neuroimage 163, 437–455. doi: 10.1016/j.neuroimage.2017.09.012, PMID: 28916180

[ref13] Liégeois R. Li J. Kong R. Orban C. Van De Ville D. Ge T.. (2019). Resting brain dynamics at different timescales capture distinct aspects of human behavior. Nat. Commun. 10:2317. doi: 10.1038/s41467-019-10317-7, PMID: 31127095 PMC6534566

[ref14] Liu X. Chang C. Duyn J. H. (2013). Decomposition of spontaneous brain activity into distinct FMRI co-activation patterns. Front. Syst. Neurosci. 7:101. doi: 10.3389/fnsys.2013.00101, PMID: 24550788 PMC3913885

[ref15] Macke J. H. Berens P. Ecker A. S. Tolias A. S. Bethge M. (2009). Generating spike trains with specified correlation coefficients. Neural Comput. 21, 397–423. doi: 10.1162/neco.2008.02-08-713, PMID: 19196233

[ref16] Masuda N. Islam S. Aung S. T. Watanabe T. (2024). Energy landscape analysis based on the Ising model: tutorial review. arXiv arXiv:2411.16979. 10.1371/journal.pcsy.0000039 PMC13215579 42211873

[ref17] Matsui T. Murakami T. Ohki K. (2018a). Mouse optical imaging for understanding resting-state functional connectivity in human Fmri. Commun. Integr. Biol. 11:e1528821. doi: 10.1080/19420889.2018.1528821, PMID: 30534348 PMC6284571

[ref18] Matsui T. Murakami T. Ohki K. (2018b). Neuronal origin of the temporal dynamics of spontaneous bold activity correlation. Cereb. Cortex 29, 1496–1508. doi: 10.1093/cercor/bhy045, PMID: 29522092

[ref19] Matsui T. Pham T. Q. Jimura K. Chikazoe J. (2022). On co-activation pattern analysis and non-stationarity of resting brain activity. Neuroimage 249:118904. doi: 10.1016/j.neuroimage.2022.118904, PMID: 35031473

[ref20] Matsui T. Yamashita K. I. (2023). Static and dynamic functional connectivity alterations in Alzheimer’s disease and neuropsychiatric diseases. Brain Connect. 13, 307–314. doi: 10.1089/brain.2022.0044 35994384

[ref21] Noro Y. Li R. Matsui T. Jimura K. (2022). A method for reconstruction of interpretable brain networks from transient synchronization in resting-state bold fluctuations. Front. Neuroinform. 16:960607. doi: 10.3389/fninf.2022.960607, PMID: 36713290 PMC9878402

[ref22] Nozari E. Bertolero M. A. Stiso J. Caciagli L. Cornblath E. J. He X.. (2024). Macroscopic resting-state brain dynamics are best described by linear models. Nat. Biomed. Eng. 8, 68–84. doi: 10.1038/s41551-023-01117-y, PMID: 38082179 PMC11357987

[ref23] Power J. D. Cohen A. L. Nelson S. M. Wig G. S. Barnes K. A. Church J. A.. (2011). Functional network organization of the human brain. Neuron 72, 665–678. doi: 10.1016/j.neuron.2011.09.006, PMID: 22099467 PMC3222858

[ref24] Preti M. G. Bolton T. A. Van De Ville D. (2016). The dynamic functional connectome: state-of-the-art and perspectives. Neuroimage 160, 41–54. doi: 10.1016/j.neuroimage.2016.12.061, PMID: 28034766

[ref25] Saggar M. Shine J. M. Liégeois R. Dosenbach N. U. F. Fair D. (2022). Precision dynamical mapping using topological data analysis reveals a hub-like transition state at rest. Nat. Commun. 13:4791. doi: 10.1038/s41467-022-32381-2, PMID: 35970984 PMC9378660

[ref26] Van Essen D. C. Smith S. M. Barch D. M. Behrens T. E. Yacoub E. Ugurbil K.. (2013). The Wu-Minn human connectome project: an overview. NeuroImage 80, 62–79. doi: 10.1016/j.neuroimage.2013.05.041, PMID: 23684880 PMC3724347

[ref27] Vidaurre D. Smith S. M. Woolrich M. W. (2017). Brain network dynamics are hierarchically organized in time. Proc. Natl. Acad. Sci. USA 114, 12827–12832. doi: 10.1073/pnas.1705120114, PMID: 29087305 PMC5715736

[ref28] Watanabe T. Hirose S. Wada H. Imai Y. Machida T. Shirouzu I.. (2014). Energy landscapes of resting-state brain networks. Front. Neuroinform. 8:12. doi: 10.3389/fninf.2014.00012, PMID: 24611044 PMC3933812

[ref29] Watanabe T. Rees G. (2017). Brain network dynamics in high-functioning individuals with autism. Nat. Commun. 8:16048. doi: 10.1038/ncomms16048, PMID: 28677689 PMC5504272

[ref30] Yeh F.-C. Tang A. Hobbs J. P. Hottowy P. Dabrowski W. Sher A.. (2010). Maximum entropy approaches to living neural networks. Entropy 12, 89–106. doi: 10.3390/e12010089

