# Supplementary figures and images for: Surrogate data analyses of the energy landscape analysis of resting-state brain activity
Source: Front Neural Circuits. 2025 Mar 14;19:1500227. doi: 10.3389/fncir.2025.1500227 (PMC11949950; doi:10.3389/fncir.2025.1500227)

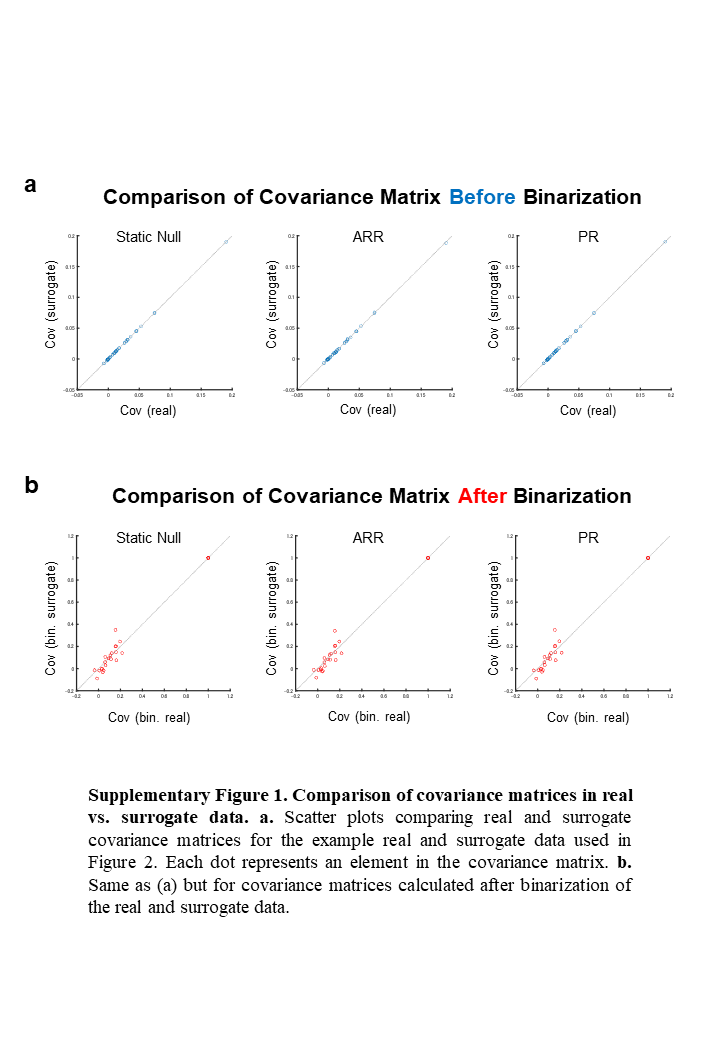

Supplement: Supplementary file 1 [file Image_1.tif]

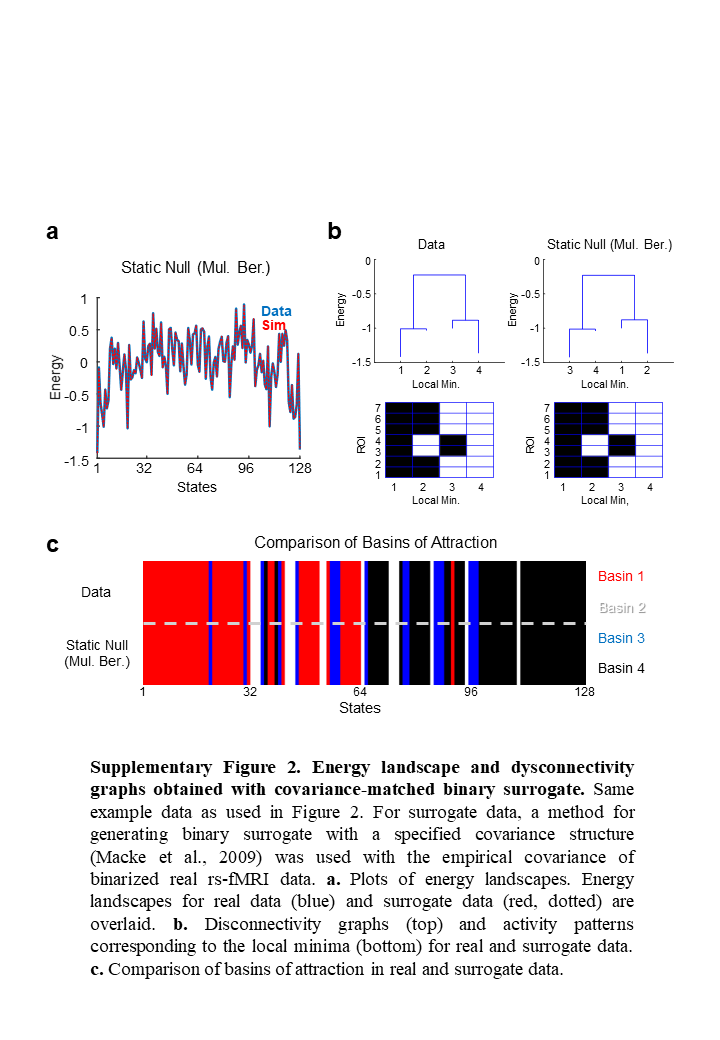

Supplement: Supplementary file 2 [file Image_2.tif]

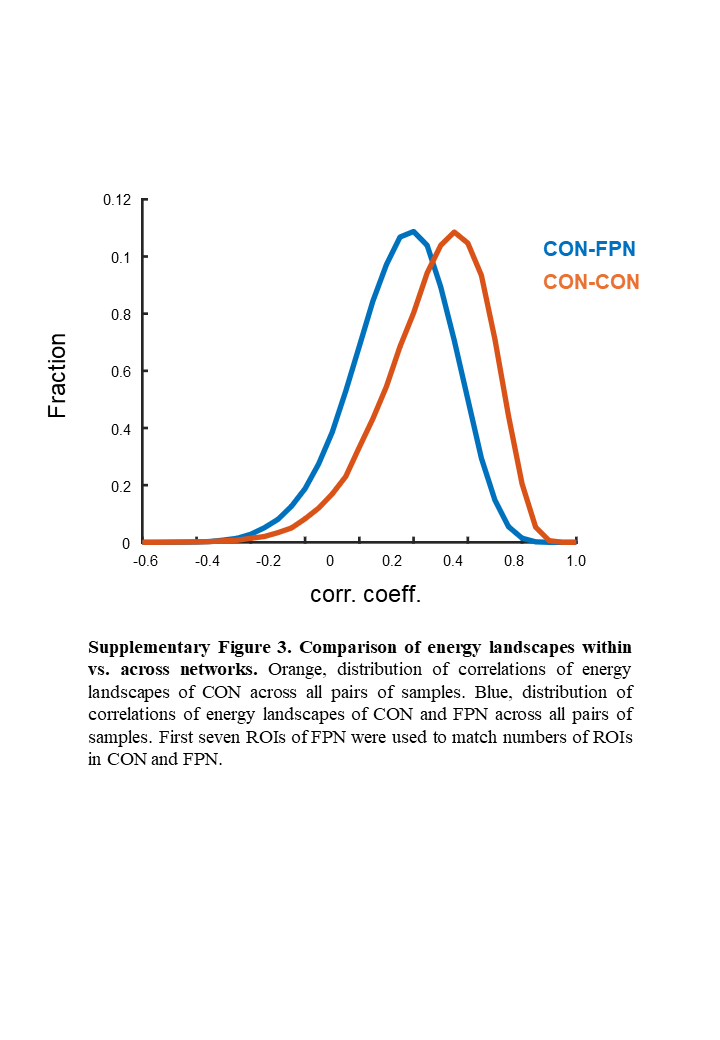

Supplement: Supplementary file 3 [file Image_3.tif]

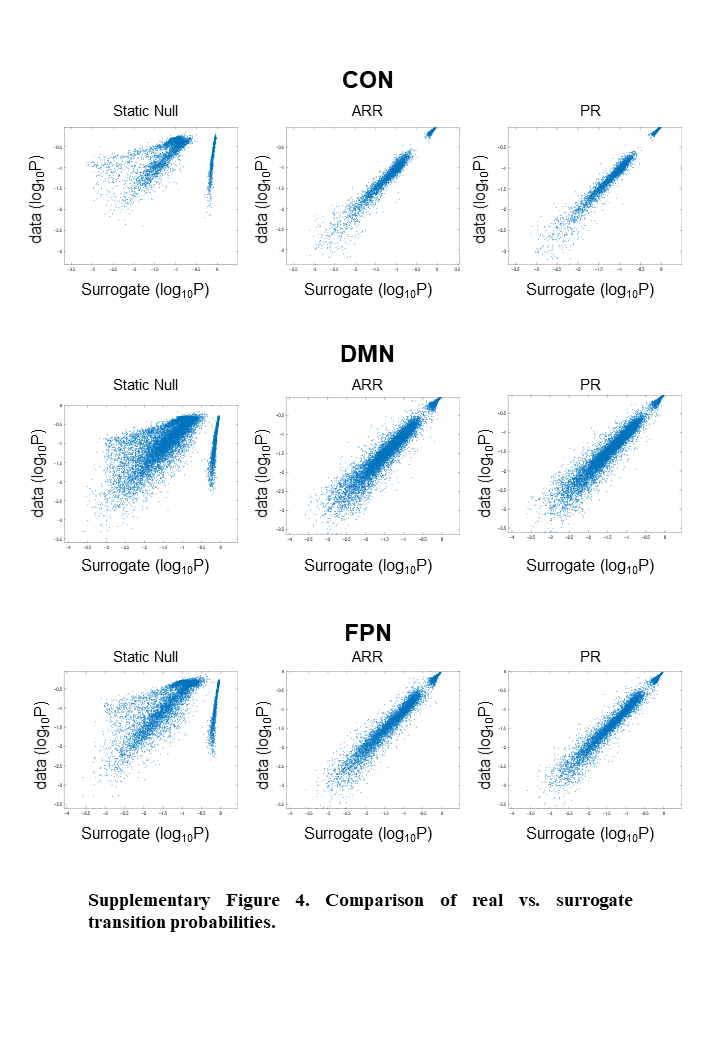

Supplement: Supplementary file 4 [file Image_4.tif]
